# Breadth of knowledge vs. grades: What best predicts achievement in the first year of health sciences programmes?

**DOI:** 10.3352/jeehp.2012.9.7

**Published:** 2012-05-16

**Authors:** Boaz Shulruf, Meisong Li, Judy McKimm, Melinda Smith

**Affiliations:** 1 Centre for Medical and Health Sciences Education, Faculty of Medical and Health Sciences, University of Auckland, Auckland, New Zealand.; 2 College of Medicine, Swansea University, Swansea, UK.

**Keywords:** Nursing students, Pharmacy students, Students, premedical, College admission test, Educational measurement

## Abstract

This study aimed to identify those features within secondary school curricula and assessment, particularly science subjects that best predict academic achievement in the first year of three different three-year undergraduate health professional programmes (nursing, pharmacy, and health sciences) at a large New Zealand university. In particular, this study compared the contribution of breadth of knowledge (number of credits acquired) versus grade level (grade point average) and explored the impact of demographic variables on achievement. The findings indicated that grades are the most important factor predicting student success in the first year of university. Although taking biology and physics at secondary school has some impact on university first year achievement, the effect is relatively minor.

## INTRODUCTION

In 2002, the New Zealand Qualification Authority (NZQA) introduced a new secondary school assessment system: the National Certificate of Educational Achievement (NCEA). Prior to this, the secondary school assessment system included the School Certificate and New Zealand Bursary, both of which were norm-based examination systems where a 'learner's achievement was ranked against outcomes of others in a group' [1]. The change in the secondary school qualification system from a norm- to standards-based system was a major move, which had wide-ranging implications across New Zealand's secondary and tertiary education sectors [2-4]. One consequence was the need for university admission processes to be reformed in order to meet the requirements of the new system; however, no empirical evidence was available to inform changes in selection processes [5].

The first NCEA cohort completed their schooling in 2004, and to date, only a small number of studies have been published on the impact of NCEA results on university achievement [6-10]. These studies, particularly those by Shulruf et al. [6, 9, 10], suggest that the quality of learning (i.e., the grades achieved in NCEA exams) has a higher predictive power for student achievement at university than the number of credits acquired [for details see: 6], i.e., the breadth of learning. This is consistent with other studies on the relationship between grades and subsequent achievement in tertiary education [11-15]. In particular, it is noted that achievement in some subjects (e.g., mathematics, physics, and the natural sciences) has a greater predictive power for university first year achievement in general than does that of subjects in the humanities and social sciences [14]. There is little published research on the impact of breadth of knowledge on student achievement. Indeed, the only systematic review on the impact of secondary school course-taking on university achievement found a low effect size (r=0.24) [9].

Universities in New Zealand invest major resources, in terms of time and effort, in selecting and training the most appropriate students in nursing, pharmacy, and health sciences programmes. However, no study has yet been published that explores the impact of breadth of knowledge (number of credits gained) as well as grade level achieved (e.g., subject grades, grade point average [GPA]) in secondary school on student achievement in these programmes, let alone within the context of the NCEA, New Zealand's unique secondary school assessment system.

The overarching aim of this study is to identify features within the secondary school qualification system that best predict achievement in three selected undergraduate professional health science programmes at the University of Auckland (UoA) in New Zealand. These programmes are: bachelor in health sciences (BHSC), bachelor in pharmacy (BPHAR), and bachelor in nursing (BNURS).

A number of studies have explored factors predicting academic achievement and progression in nursing and pharmacy programmes and concluded that pre-admission GPA or high pre-university academic achievement is a good predictor for early academic achievement in professional programmes [16, 17]. This has also been supported by other studies focussing on medicine [18-20].

Subjects taken and achievement in these subjects are also important indicators of academic achievement in the early years of professional programmes. Many studies indicate that pre-admission high grade achievement in biology, mathematics, and another science subject predicts academic achievement in the early parts of the programmes (where curricula tend to be structured around biomedical or natural sciences). However, this effect levels out as students progress through their studies, and in particular as students move into clinical areas in the later stages of a degree, or professional practice following graduation [21-23]. The pre-admission GPA is therefore much less relevant as a predictor of professional performance and overall capability than of academic (knowledge-based) achievement. Internationally, many health professional programmes have moved away from reliance on academic performance (as typified by pre-admission GPA or equivalent) as the only, or key, measure of aptitude prior to admission. Most professional programmes include interview or performance tests and increasingly offer specific testing programmes designed for individual professions, such as the Test of Essential Academic Skills (TEAS), a nursing aptitude test used in the United States [17] or the multiple mini-interview (MMI). Newton et al. [17] suggested that both scholastic aptitude (as measured by previous GPA) and nursing aptitude (as measured by TEAS) are important to predict later performance and completion of nursing programmes.

The study by McCall et al. [21] on the influence of admission tests on Pharmacy Licensing Examination scores concluded that the pre-pharmacy GPA had a positive but low correlation with subsequent performance on the licensing examination. However, GPA was only one of a number of admission variables that correlated with the licensing examination score, and the capability of these admission variables to predict performance was relatively low. McCall et al. [21] suggest that "completion of a four year professional degree pharmacy program ultimately 'levels the field'". A systematic review conducted by Ferguson et al. [24] examining data on the predictive validity of factors believed to be significant predictors of success in medicine also found that on average previous academic performance accounted for 23% of the variance in undergraduate achievement and 6% of the variance in postgraduate competence.

The New Zealand's NCEA is unique in providing highly detailed information on student achievement [25, 26]. In particular, it provides information on the breadth of knowledge that is measured by the number of credits students have acquired since credits are given to unit or achievement standards (described below) that each student successfully obtains. The standards normally cover discrete topics and students are not permitted to sit for the examinations of two or more overlapping topics [26]. A student passing an assessment relating to any NCEA standard indicates that they have reached a threshold of knowledge and competence in a particular topic. However, the level of competency cannot be identified by measuring the number of credits; the level of competency can only be measured by the grade achieved [6].

Robust models for the analysis of NCEA results have recently been developed by Shulruf et al. [6, 10, 26]. The study reported in this paper uses their [26] definition of NCEA GPA to measure quality of learning and takes the analysis a significant step forward by looking at the impact of achievement in individual subjects on three discrete academic programmes. This study illuminates some important features of the NCEA in relation to predicting future students' success in nursing, pharmacy and health science programmes.

It is acknowledged that students' academic achievement in their first two years at university is a complex mix of factors. These relate not only to individual ability, learning styles and preferences, motivation and engagement with the courses on offer, but also to the relationship between the content, conceptualisation of knowledge, understanding and level of achievement in 'subjects' taken via NCEA, and the way in which these 'subjects' are presented, taught and assessed at university. This study, however, focuses only on the relationships between achievement in NCEA (grades, and number of credits gained) and subsequent academic achievement (as measured by GPA) in the first year of university.

The NCEA includes two types of standards: 'unit standards' (US) and 'achievement standards' (AS). Programmes taught in schools can be assessed using either US or AS, or a combination of both. For each AS, a student can achieve any of four levels of achievement: 'not achieved', 'achieved', 'merit' or 'excellence'. US, on the other hand, offer only two levels of achievement: 'not achieved' or 'achieved'. Both standards are usually offered at levels 1, 2, and 3, depending upon the degree of difficulty. However, US achievements, particularly in vocational subjects, may go beyond level 3. Typically, but not necessarily, level 1 standards are taught in Year 11, level 2 in Year 12, and level 3 in Year 13 (last year of secondary school). Each standard achieved is worth a prescribed number of credits, usually reflecting the number of hours a student would be expected to study to complete the standard [26].

With the introduction of the NCEA, NZQA also introduced a system of grade averages based on the grade achieved for each AS or US [26]. Assigning an overall grade average for NCEA outcomes assists with university admission decisions for 'limited entry' tertiary qualifications (where there is strong competition among applicants for a limited number of places). In calculating the grade average, "not achieved" scores zero; "achieved" scores two; three is assigned to 'merit'; and four is allocated to 'excellence' [26]. At the UoA, for example, applicants are ranked according to the grade average of their best 80 credits at level 3 or higher, over a maximum of five approved subjects. In addition, students applying for certain programmes (particularly those with limited entry numbers such as medicine and health professional programmes) may be required to have credits in specific subjects and/or to have a threshold GPA which is higher than the baseline for university entrance (Table 1).

## MATERIALS AND METHODS

The analysis in this study used NCEA results for subjects on the University's 'approved subjects' list only (Table 1). The structure of the NCEA distinguishes between the breadth of knowledge acquired (represented by the number of credits gained) and the level of knowledge (represented by the NCEA subject GPA - the weighted mean of the grades of all standards students take within each subject). Theoretically, these two variables are independent although the correlation between these variables (for the overall NCEA GPA and overall number of credits) is relatively high (r=0.67). Other variables included in the analysis are gender, ethnicity (ethnicities are defined as Pākehā [NZ European], Māori [the NZ indigenous people], Pacific Islanders, Asians, and others) and school decile - an index for the socioeconomic status (1 is low and 10 is high) of the school's student population. In this study, private schools were included as decile 11 since most of them are very selective and typically include students from more affluent families.

Administrative data relating to 245 students who sat for the NCEA level 3 (L3) examination in 2004 and subsequently studied first year programmes in the BPHAR (n=72; females 76% ), BNURS (n=49; females 88%), and BHSC (n=124; females 64%) were used in this study. Student distribution by programme and ethnicity is presented in Table 2. Based on the literature reviewed (cited in the introduction) we assumed that secondary school experiences, particularly learning outcomes, affect learning outcomes at the university, and hence used a linear regression model. The independent variables were demographic variables (gender, ethnicity, and school socioeconomic decile) and NCEA achievements (credits gained and NCEA GPA per subject). The dependent variable was the student's end of first year UoA GPA in these programmes.

Linear regression models were employed to identify the predictors of student UoA GPA. The regression models employed two blocks. The first block included demographic variables, including the programmes studied at the university ('enter method' i.e., all variables entered at once); and the second block included subject NCEA GPA and number of credits of all NCEA subjects ('stepwise method'). The stepwise method is preferable for such an exploratory investigation and has been commonly employed in similar research [22, 27-29].

The NCEA comprises thousands of learning standards across tens of subjects [26]; therefore, there are a large number of missing values for the NCEA subject GPA and credit numbers that could jeopardise any regression analysis. To overcome this challenge and enable us to measure the impact of secondary school learning, we assumed that the contribution of a subject to student knowledge was similar if the student achieved a fail (NCEA subject GPA, 0) or did not study that subject.

## RESULTS

The overarching aim of this study is to identify those features of the NCEA that best predict achievement (as measured by UoA GPA) in three selected health professional programmes (BHSC, BPHAR, and BNURS). In particular, this study focuses on two specific objectives: 1) to compare the contribution of breadth of knowledge and grades to achievement in these undergraduate programmes; and 2) to identify the impact of demographic variables on achievement in these programmes.

**Fig. 1:**
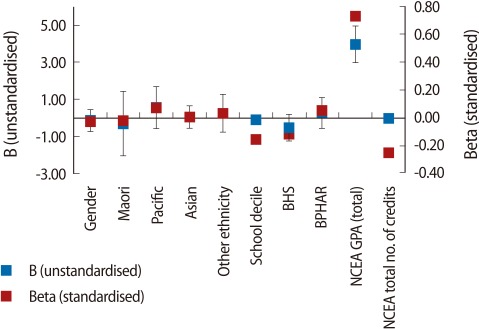
Linear regression, all students (summary NCEA; stepwise method). B (unstandardised), Beta (standardised) coefficients. BHSC, bachelor in health sciences; BPHAR, bachelor in pharmacy; NECA, National Certificate of Educational Achievement; GPA, grade point average.

The first regression model looked at the total number of students in the study across all programmes. The model included all variables of all students from all programmes along with students' summative results of the NCEA (total NCEA credits acquired and overall NCEA GPA achieved) (Table 3, Fig. 1). The results show that, with the exception of school decile (which had a small negative effect, i.e., the higher the decile the lower the first year GPA) (Tables 3, 4), demographic variables did not affect first year UoA GPA. In addition, although there were differences in NCEA achievement between students entering different programmes, the difference in first year UoA GPA across programmes (BHSC, BNURS, and BPHAR) was insignificant. However, the largest impact on UoA GPA was the NCEA GPA (beta, 0.73). The number of credits acquired, however, had a smaller, but negative, impact (beta, -0.25).

**Fig. 2:**
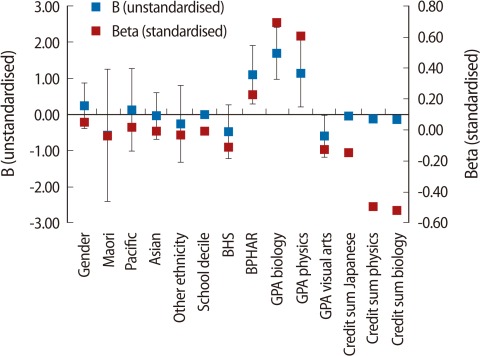
Linear regression, all students (individual subjects; stepwise method). B (unstandardised), Beta (standardised) coefficients. Creadit sum is summary of credits earned in a field of study. GPA, grade point average; BPHAR, bachelor in pharmacy; BNURS, bachelor in nursing; BHSC, bachelor in health sciences.

To identify the impact of individual subjects, the next analysis included a similar linear regression model that included the same variables. However, in this case the summative results of the NCEA results on each subject (NCEA subject GPA and subject number of credits) were included in the model using the Stepwise method (Table 4, Fig. 2). The results were interesting on two fronts: 1) that demographic variables had no effect on student first year UoA GPA and 2) that only biology and physics subjects had a positive effect on the overall first year UoA GPA, and that the impact came from the NCEA GPA rather than the number of credits.

**Fig. 3:**
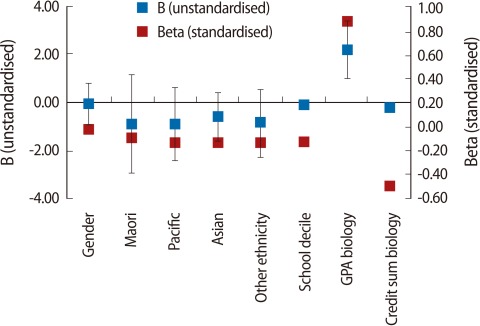
Linear regression bachelor in health sciences (BHSC). B (unstandardised), Beta (standardised) coefficients. GPA, grade point average.

**Fig. 4:**
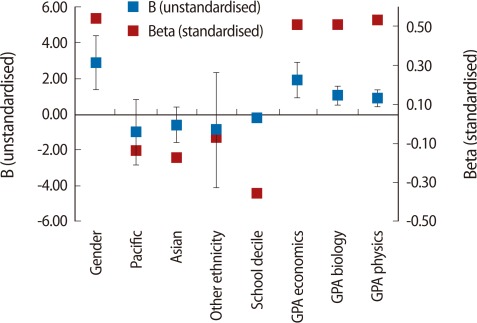
Linear regression bachelor in nursing (BNURS). B (unstandardised), Beta (standardised) coefficients. GPA, grade point average.

**Fig. 5:**
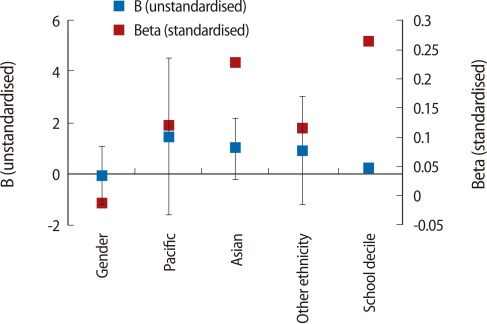
Linear regression bachelor in pharmacy (BPHAR). B (unstandardised), Beta (standardised) coefficients.

**Fig. 6:**
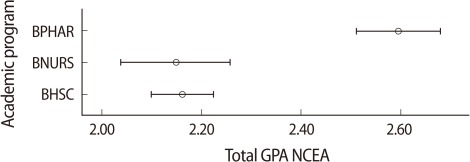
Grade point average (GPA) of the National Certificate of Educational Achievement (NCEA) by university academic programmes. BPHAR, bachelor in pharmacy; BNURS, bachelor in nursing; BHSC, bachelor in health sciences.

To explore this further, similar regression models were separately used for students in each programme. The results (Figs. 3-5; regression tables are not presented) demonstrate that, with the exception of school decile, which had a significant positive impact only on BPHAR students (Fig. 5), demographic variables did not have a significant impact on students' first year UoA GPA. In terms of subjects, none of the subjects had any impact on BPHAR first year UoA GPA. The NCEA GPA in biology had a positive and significant impact on first year UoA GPA for the BHSC students; however, the impact of the number of credits acquired in biology was negative (Fig. 3). For students in the BNURS programme, NCEA GPA in biology, physics, and economics had a positive and significant impact (Fig. 4). It is also noted that the only significant difference in the independent variables across the programmes was in their total NCEA GPA (P=0.001), where BPHAR students had significantly higher NCEA GPA than their BNURS and BHSC counterparts (Fig. 6).

## DISCUSSION

The objectives of this study were twofold: 1) to compare the contribution of breadth of knowledge and grades to the success in three undergraduate health professional programmes (BHSC, BNURS, and BPHAR) and 2) to identify the impact of demographic variables on achievement in these programmes. In terms of the first objective, the most important finding was that grades had a significant positive impact on student achievement in these programmes while breadth of knowledge had no impact at all. Although it has previously been shown that the size effect of the impact of courses taken at secondary school on tertiary education achievement appears to be relatively low (r=0.24) [9], research exploring the impact of specific secondary school knowledge on university achievement could not be identified in the literature. However, the impact of secondary school grades and aptitude tests on university achievement has been thoroughly investigated and indicates a moderate association [15, 22]. The findings of this study provide a unique insight into this issue, suggesting that, while the GPA does make a difference, the number of credits gained in school, that is, breadth of knowledge, made little difference in terms of achievement in three undergraduate health profession programmes.

It should be noted that national university entrance in New Zealand and the admissions criteria for these programmes in particular specifies some subjects as mandatory; therefore, students do not gain entry with a free choice of subjects, as they might in other countries [26]. The UoA's admission criteria for the three programmes investigated in this study require students to strategically navigate their studies through the secondary school curriculum and assessment system (NCEA). However, many fail to manage this, particularly in lower decile schools, where career advice or parental guidance regarding university entrance to specific programmes may be less available to pupils [6, 7, 30, 31]. Selecting students on their NCEA GPA, which emphasises the grade level of achievement in secondary school, however, might not only improve the student selection process, in terms of reflecting students' potential achievement in the health professions programmes, but may also address important equity issues and enable more students from under-represented groups to enter and succeed in these programmes. This could ultimately reduce the need for special or affirmative action admission policies [6, 10].

A second implication stemming from objective [1] relates to decisions made while students are still studying at secondary school and their ability to overcome potential barriers to success, mainly subject choice, which is an important factor affecting their eligibility for admission. The results of this study suggest that, irrespective of subjects taken and breadth of knowledge acquired, once students have been admitted to university, it is their secondary school grades (i.e., levels of achievement) that have the greatest impact on their achievement in the first year of university study (Tables 3, 4, Fig. 1). Thus, level (rather than breadth of knowledge) has a greater impact on university GPA. However, it is noted that the NCEA GPA in biology, and to a lesser extent in physics, had the greatest impact on university GPA in BHSC and BNURS, but not in BPHAR. This finding is notable as it suggests that level of knowledge, if relevant to the undergraduate programme, impacts student achievement. Consequently, if breadth of knowledge has a negligible impact on achievements (Tables 3, 4, Fig. 1), it is suggested that a key factor for success in BNURS and BHSC programmes is the achievement of high grades in biology and physics.

The challenge of making the right decisions under current admission policies is great, and a minefield for students to negotiate [7, 30]. If current admission policies are to remain unaltered, schools need to take the lead in providing students with high-quality, fully-informed university/career advice to assist them in planning for university entry and enhance their chances of both admission and success.

It is interesting to note that achievements in the BPHAR were not affected by NCEA results (Fig. 5). An explanation for this is shown in Fig. 6, which indicates that students in BPHAR had a significantly higher NCEA GPA. This finding is important and provides additional support to the notion above relating to the importance of level of knowledge. The BPHAR findings suggest that achieving a threshold (or 'high enough') NCEA GPA may secure success in the BPHAR programme regardless of demographic factors or the number of credits achieved, as these did not have any significant impact on the university GPA. This suggests that once students achieve a threshold NCEA GPA, regardless of the subjects, they are likely to succeed in predominantly knowledge-based early university studies. As noted earlier, however, this does not predict subsequent achievement or performance in clinical or professional practice, which supports 'mixed methods' admissions policies for health professions' programmes.

The second objective of this study was to identify the impact of demographic variables on achievement in these programmes. The results indicate that ethnicity and gender did not have any significant impact on university GPA, with the exception of females, who had significantly higher achievement in the BNURS programmes than males. On the one hand, this finding suggests that the current admission processes of these three programmes allow the enrolment of students whose potential to succeed is unrelated to their socio-demographic status. On the other hand, however, it is possible that students from under-represented groups are actually under-represented among the applicants to these programmes, which ultimately affects the student population (unfortunately no data to investigate this further were available). If this is the case, it is important to find appropriate ways to enhance and address equity via secondary schools as well as through appropriate changes in the admission criteria [6].

Three caveats must be considered in interpreting the findings of this study. Since the study used data from students who had already been admitted to the programmes, there is some risk of bias relating to the restriction of range [32]. However, since undertaking a randomised controlled study is not feasible or ethical, further research on this topic, using other acceptable methodologies (e.g., quasi experimental design) is recommended, particularly if admission policies change. The second caveat relates to the differences in the admission criteria and selection methods (Table 1) across the programmes investigated in this study [33]. For example, admission procedures to the BPHAR and BNURS programmes include an interview, which is not required for admission to the BHSC programme. Unfortunately, information on the results of the interviews was not available and was therefore not included in the analysis. Hence, the impact of the interview component within the selection process on admission, and students' subsequent performance, remains unknown and might affect the results. Finally, it was not possible to investigate the influence and impact of the different programmes' curricula, teaching or learning and assessment methods on first year performance. This is another important area for further research and not within the scope of this study.

In conclusion, this study provides important insights into some previously under-researched factors affecting student success in undergraduate health professional programmes. The uniqueness of the New Zealand secondary school assessment (NCEA) has enabled an in-depth analysis of the relationship between school achievement and performance in first year university study which disentangles some of the complexities between depth and breadth of knowledge. However, the results of this study may apply to many other jurisdictions where students face similar challenges. This study clearly indicates that level of knowledge (identified by grades) is the most important factor predicting student success in the first year of university. Studying subjects in secondary schools that are relevant to the early stages of these programmes (i.e., biology or physics) is helpful; however, a breadth of achievement across different topics with mediocre grades does not predict subsequent academic success. Hence, it is suggested that policy makers, educational institutions and students work together to improve academic pathways within and beyond secondary education, particularly leading to undergraduate programmes in the health professions.

## Figures and Tables

**Table 1:**
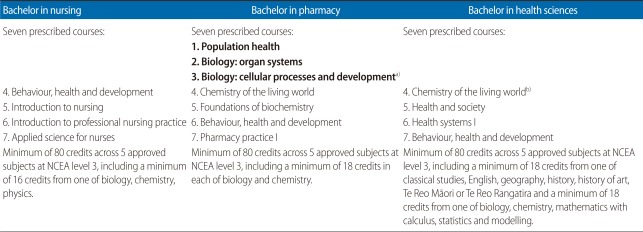
University of Auckland medical and health sciences programmes: courses and entry requirements 2005

NCEA, National Certificate of Educational Achievement. ^a)^Key to courses in Year 1: bold, courses common to all programmes. ^b)^Students not intending to enter medicine at end of Year 1 can take two other approved electives instead of 3&4.

**Table 2:**
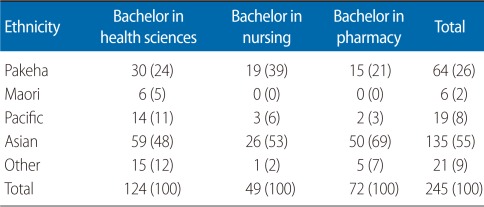
Student distribution by ethnicity and programme

Values are presented as number (%).

**Table 3:**
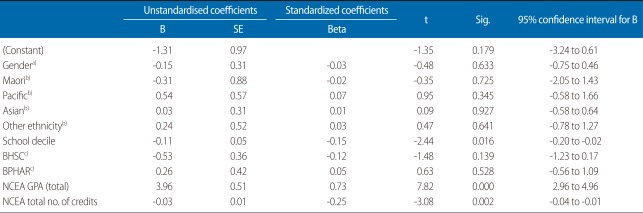
Linear regression all students (summary NCEA)

NCEA, National Certificate of Educational Achievement; SE, standard error; BHSC, bachelor in health sciences; BPHAR, bachelor in pharmacy; GPA, grade point average. ^a)^Reference, male. ^b)^Reference, European/Pakeha. ^c)^Reference, nursing.

**Table 4:**
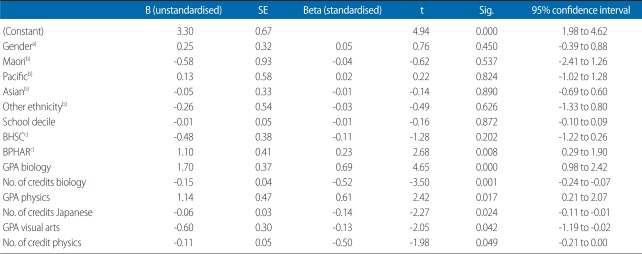
Linear regression all students all programmes (dependent variable 1st year GPA)

GPA, grade point average; SE, standard error; BHSC, bachelor in health sciences; BPHAR, bachelor in pharmacy. ^a)^Reference, male. ^b)^Reference, European/Pakeha. ^c)^Reference, nursing.
